# Supplementary material for: Loss of the KN Motif and AnKyrin Repeat Domain 1 (KANK1) Leads to Lymphoid Compartment Dysregulation in Murine Model
Source: Genes (Basel). 2023 Oct 16;14(10):1947. doi: 10.3390/genes14101947 (PMC10605996; doi:10.3390/genes14101947)

Supp. Figure S1

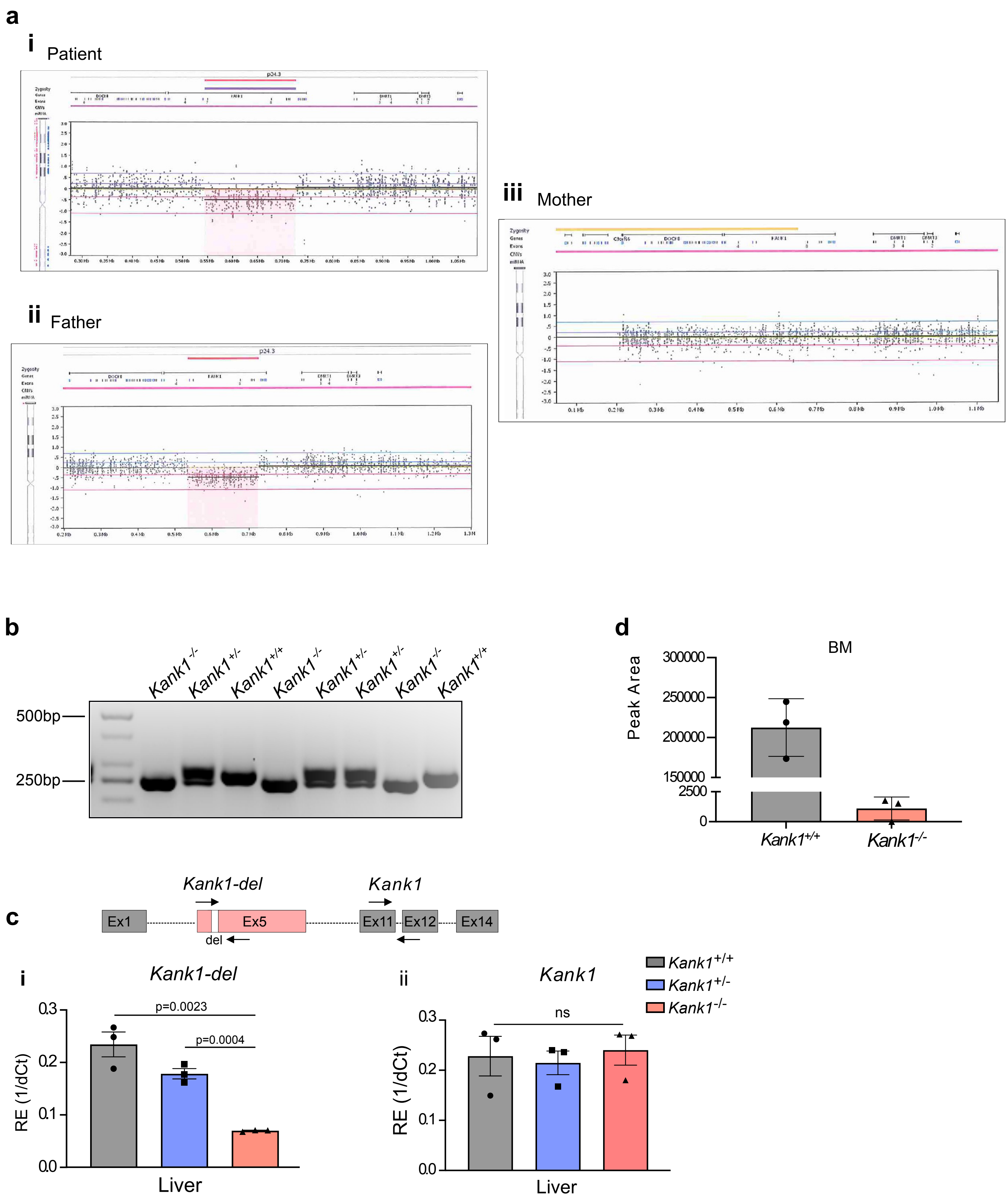

Supp. Figure S1

e

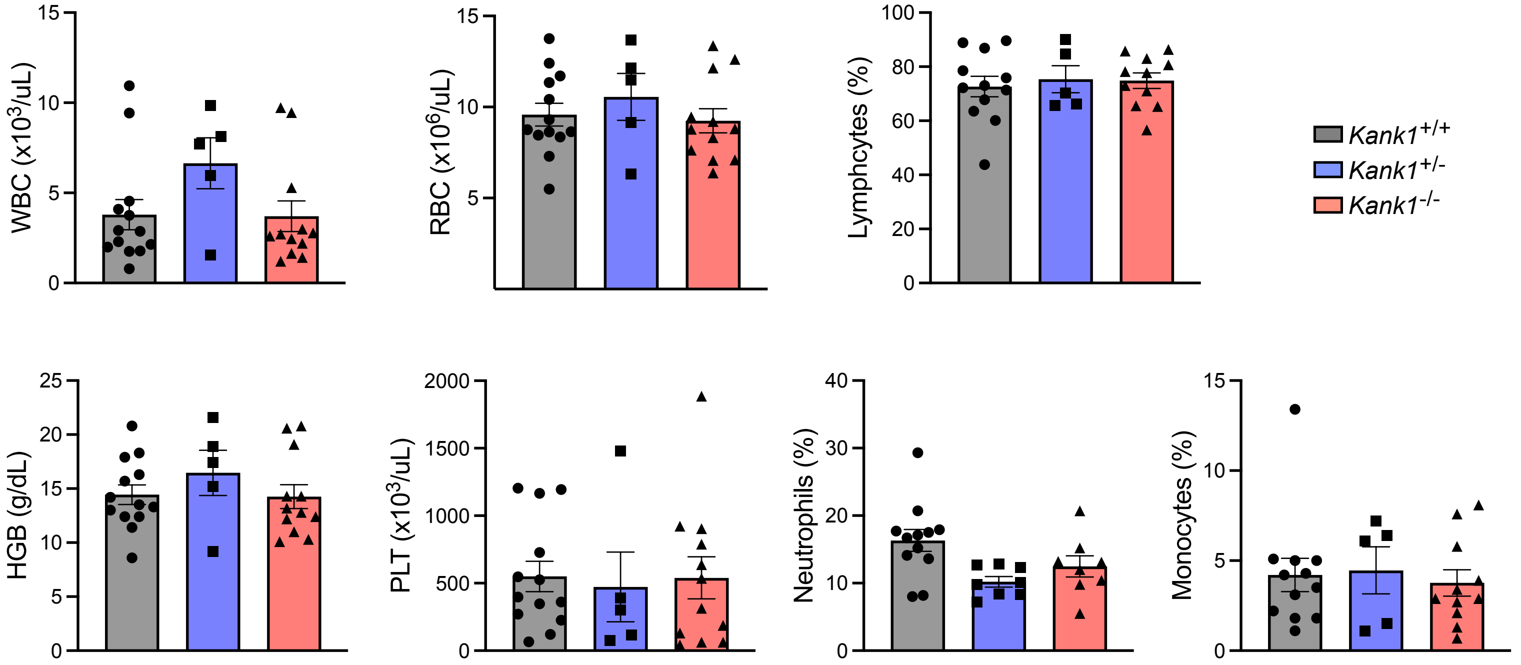

f

Percentage of HSPCs populations in BM of adult mice

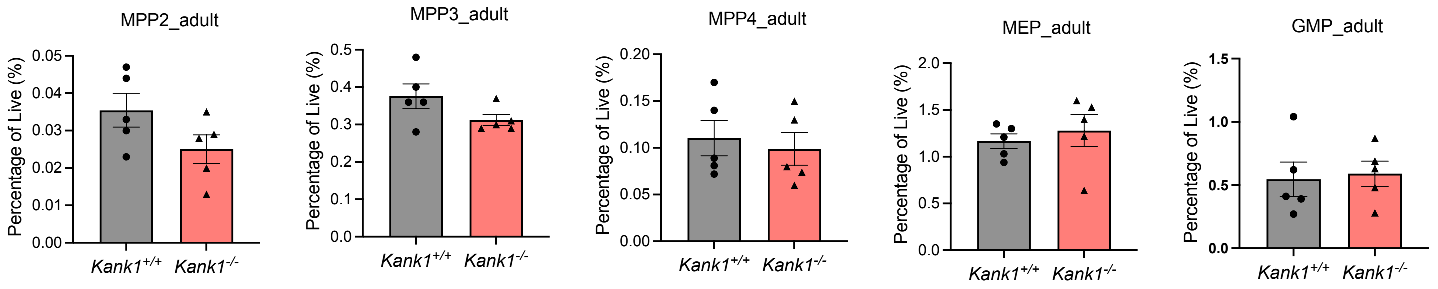

g

Percentage of HSPCs populations in BM of old mice

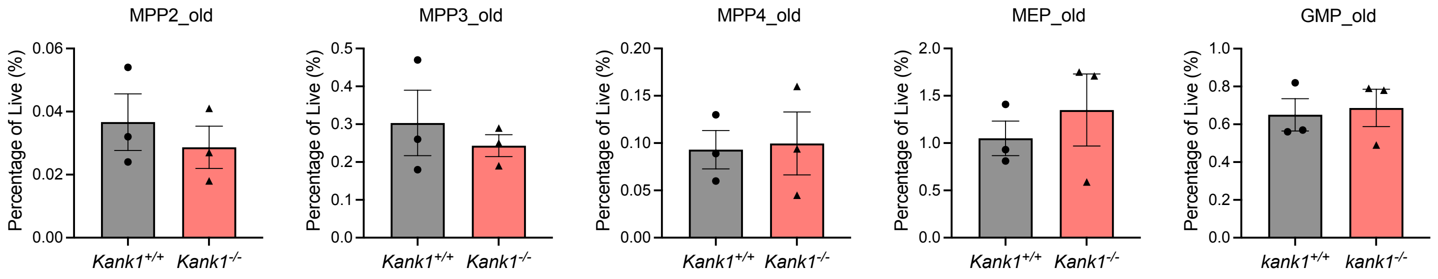

Supp. Figure S1

h

HSPCs populations gating strategy

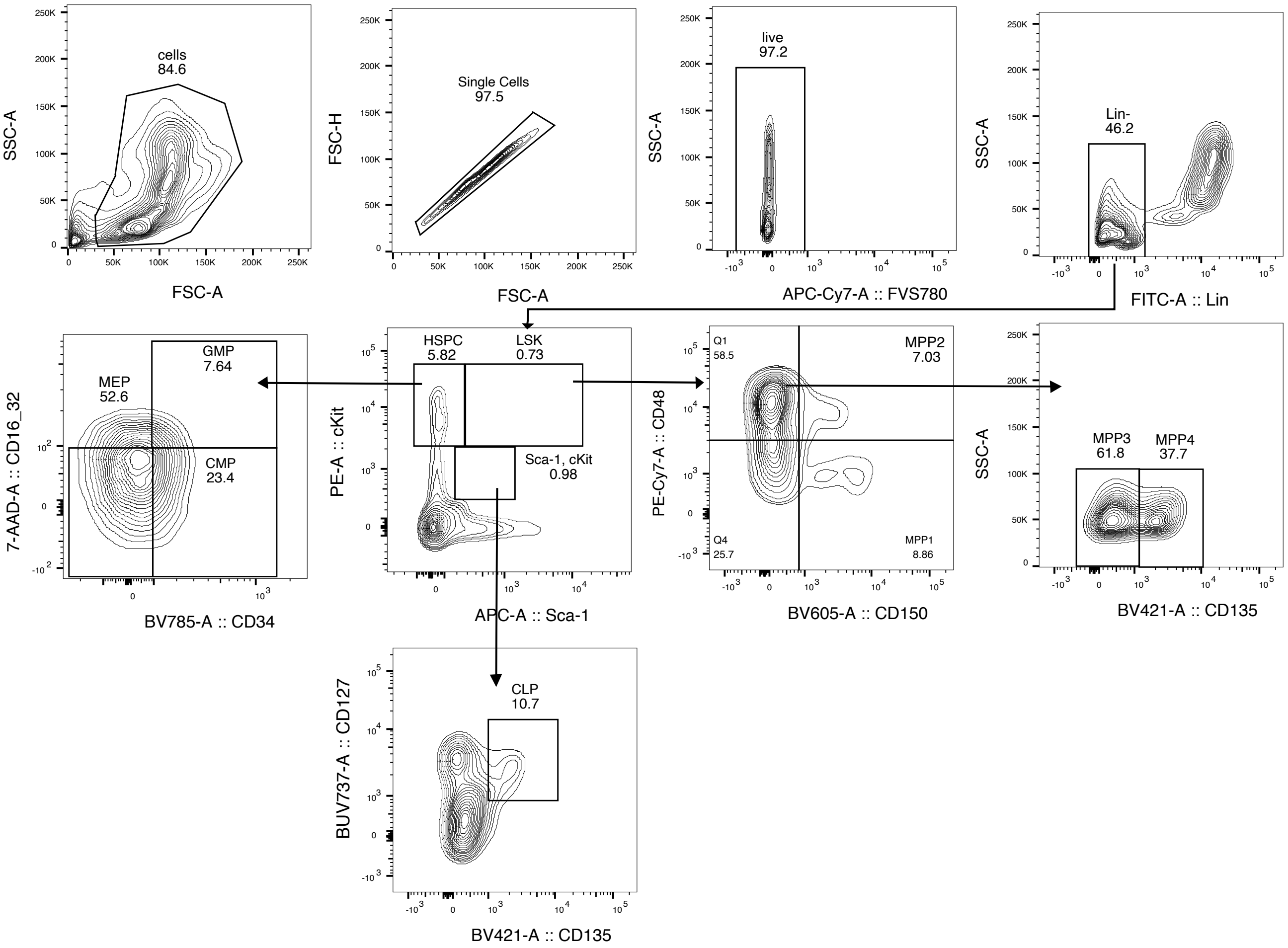

Supp. Figure S1

i

Percentage of Lineage populations in BM of adult mice

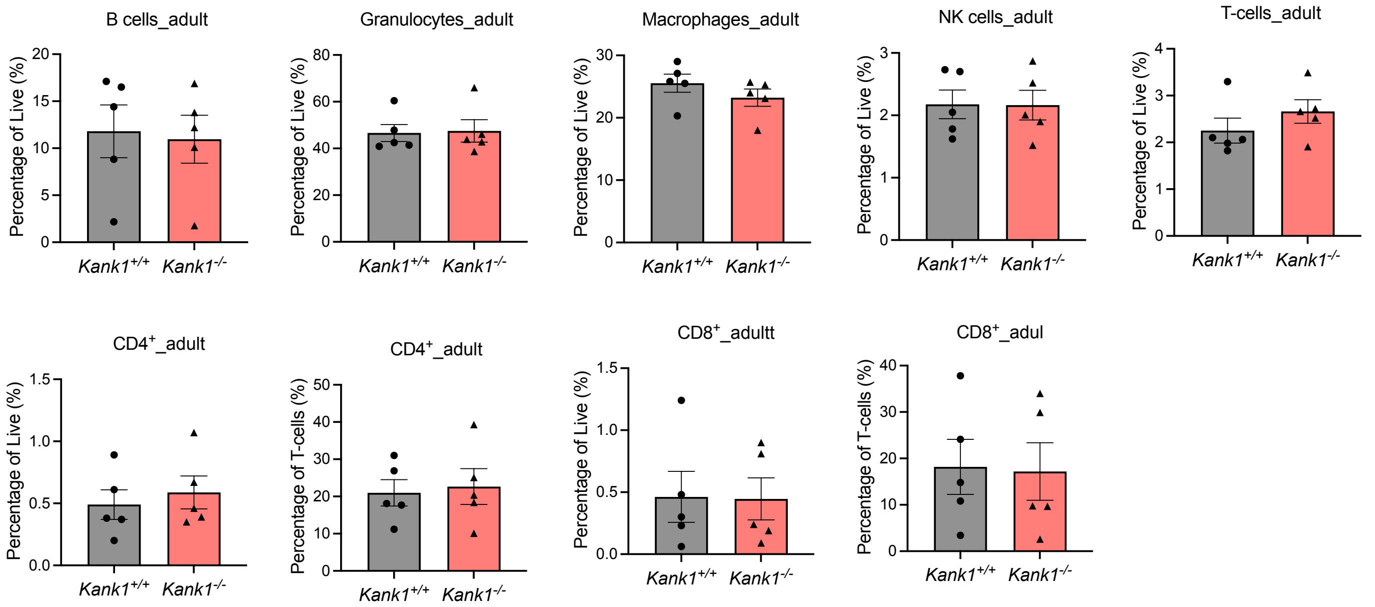

j

Percentage of Lineage populations in BM of old mice

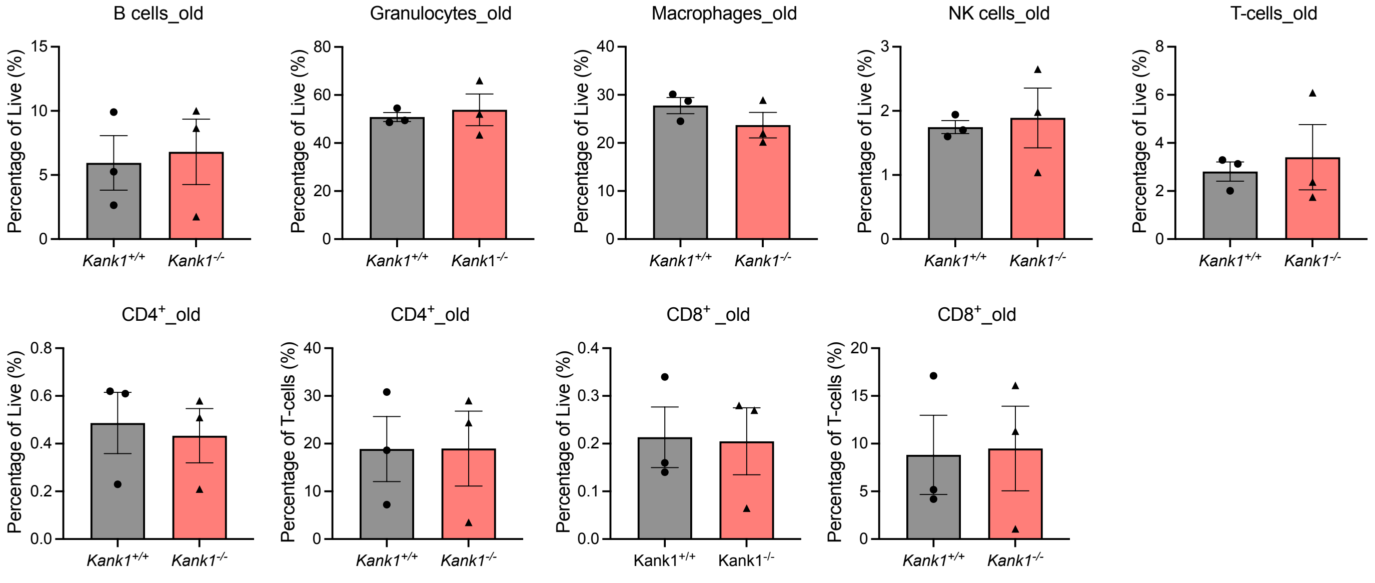

Supp. Figure S1

k

Lineage populations gating strategy

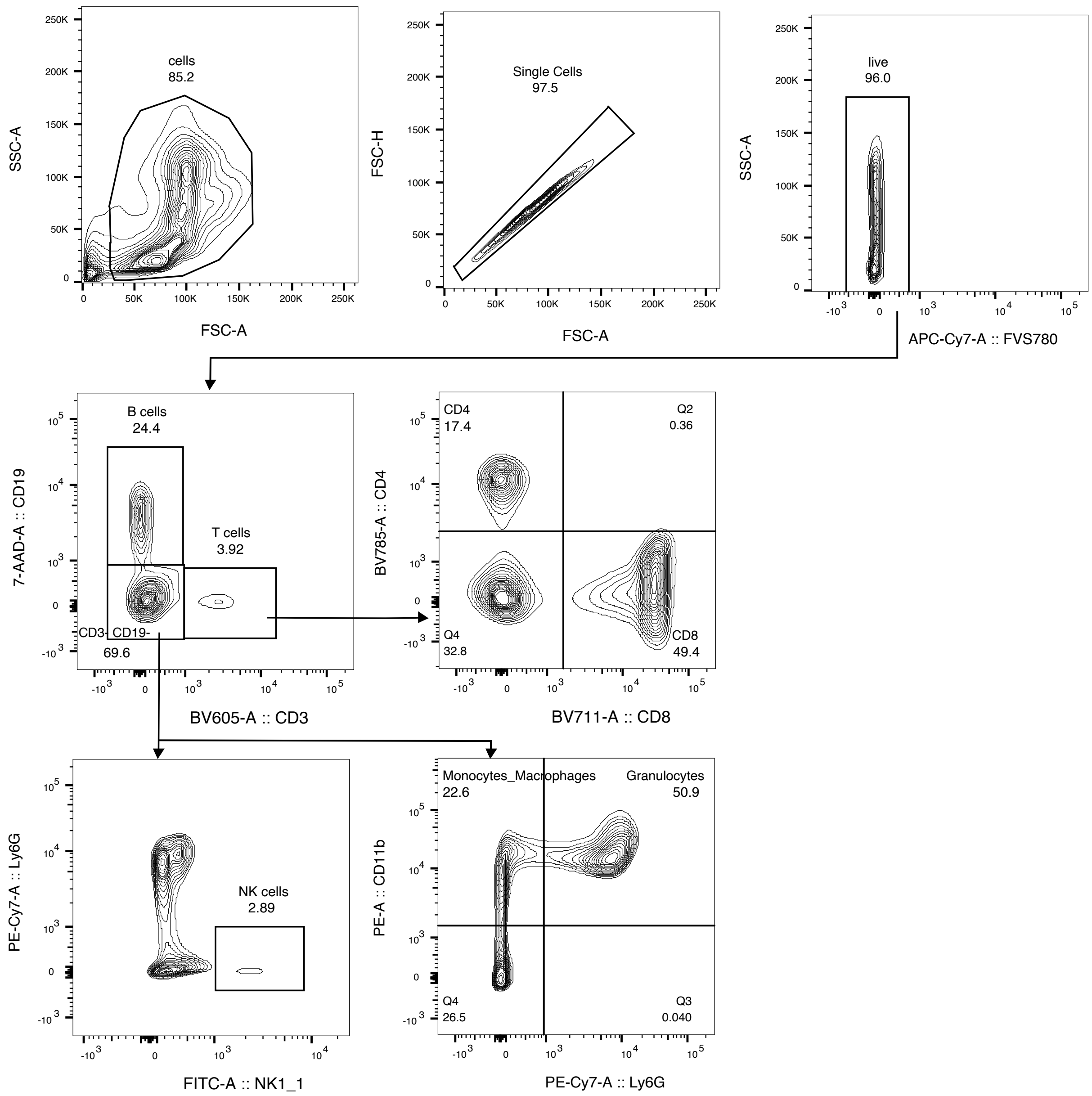

l

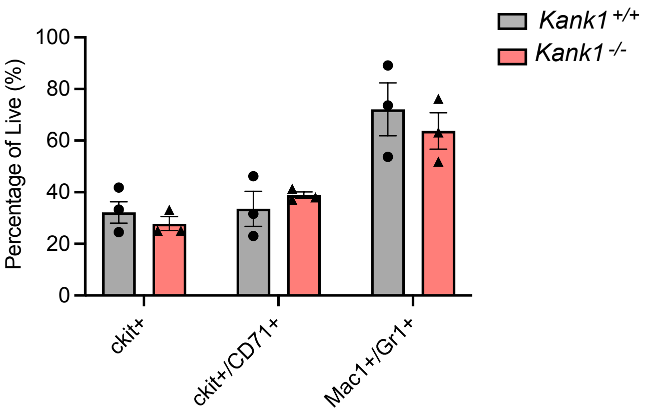

Supp. Figure S1

m

1st plating

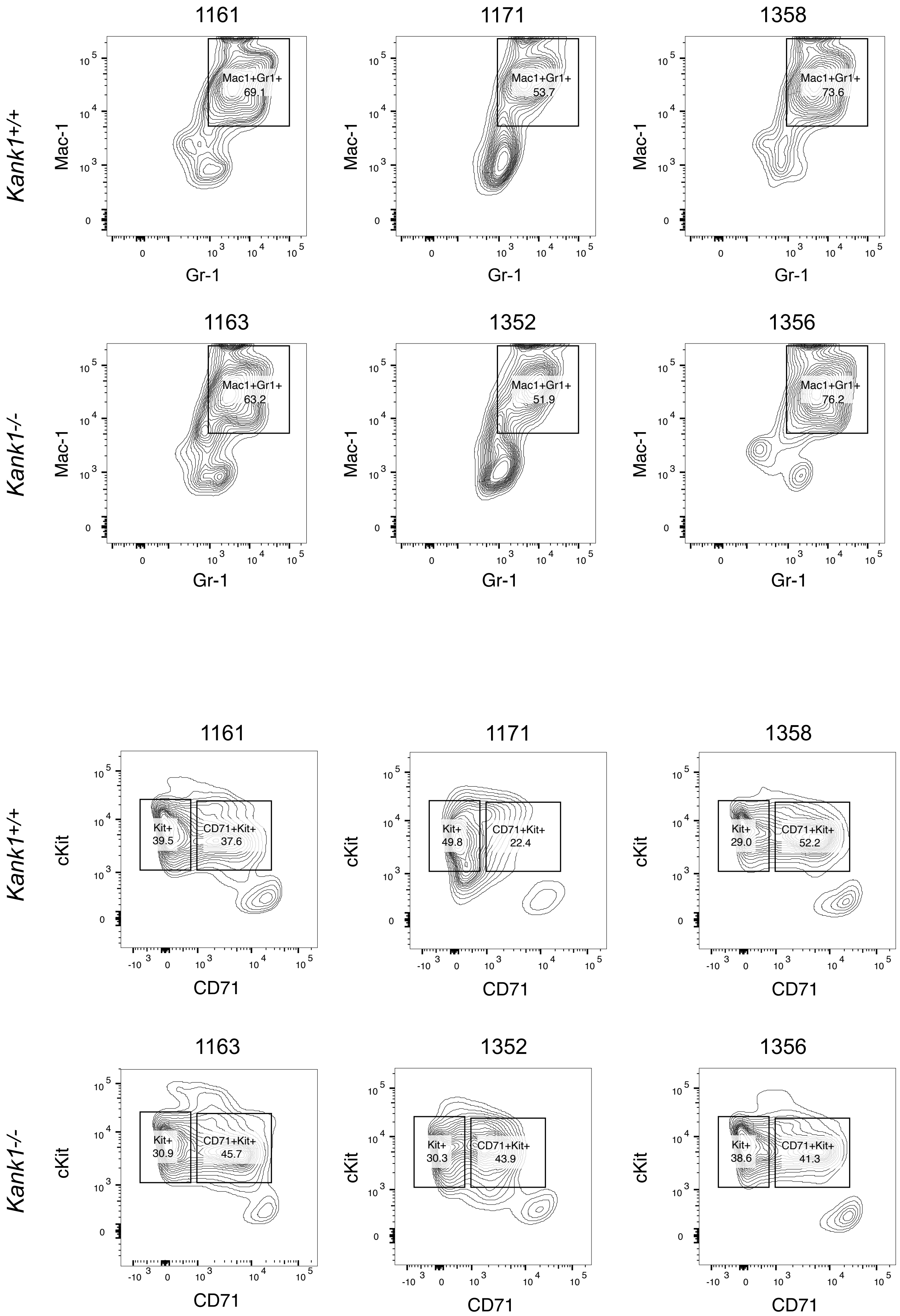

Supp. Figure S1

n

2nd plating

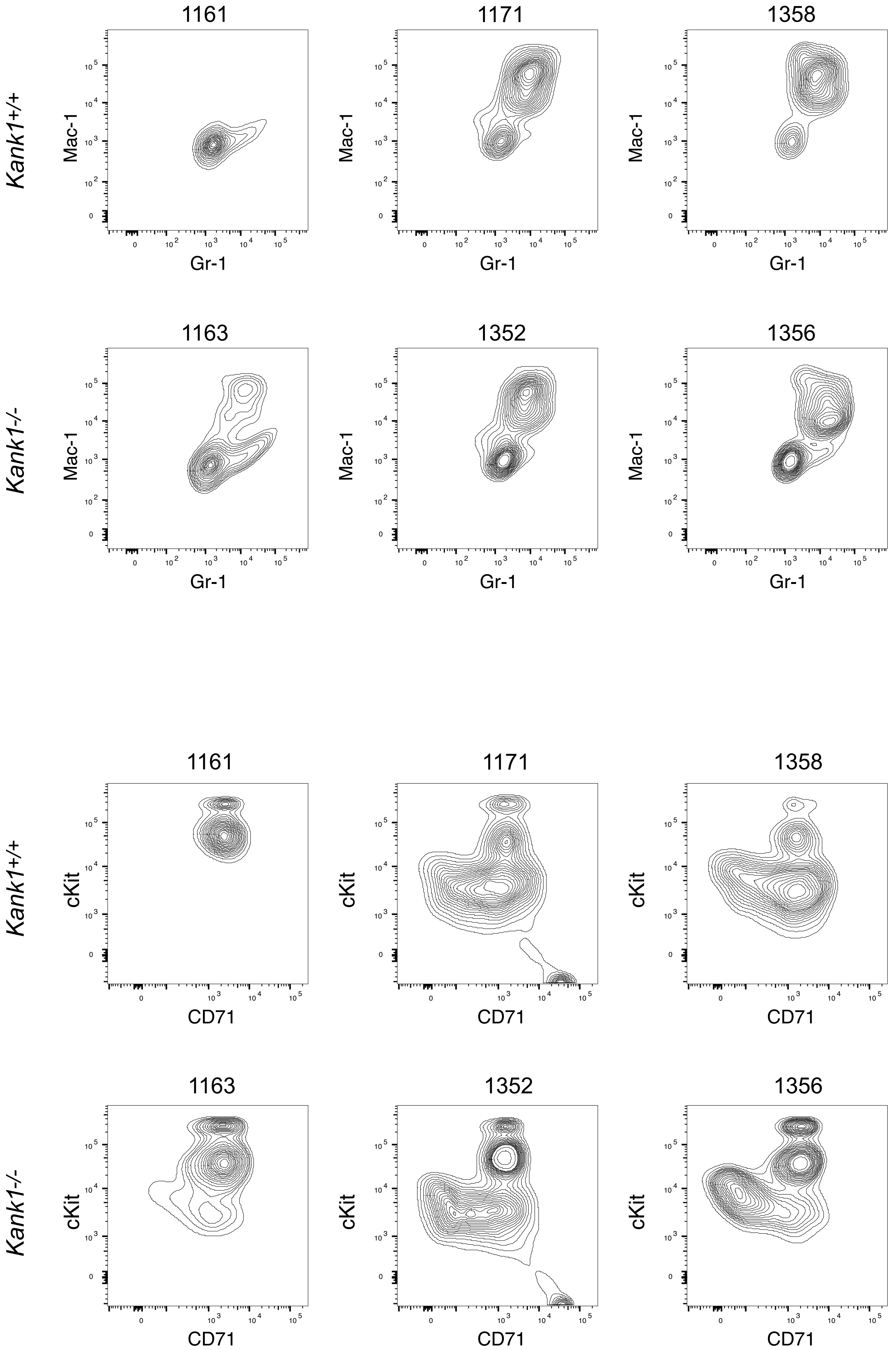

Supp. Figure S1

o

3rd plating

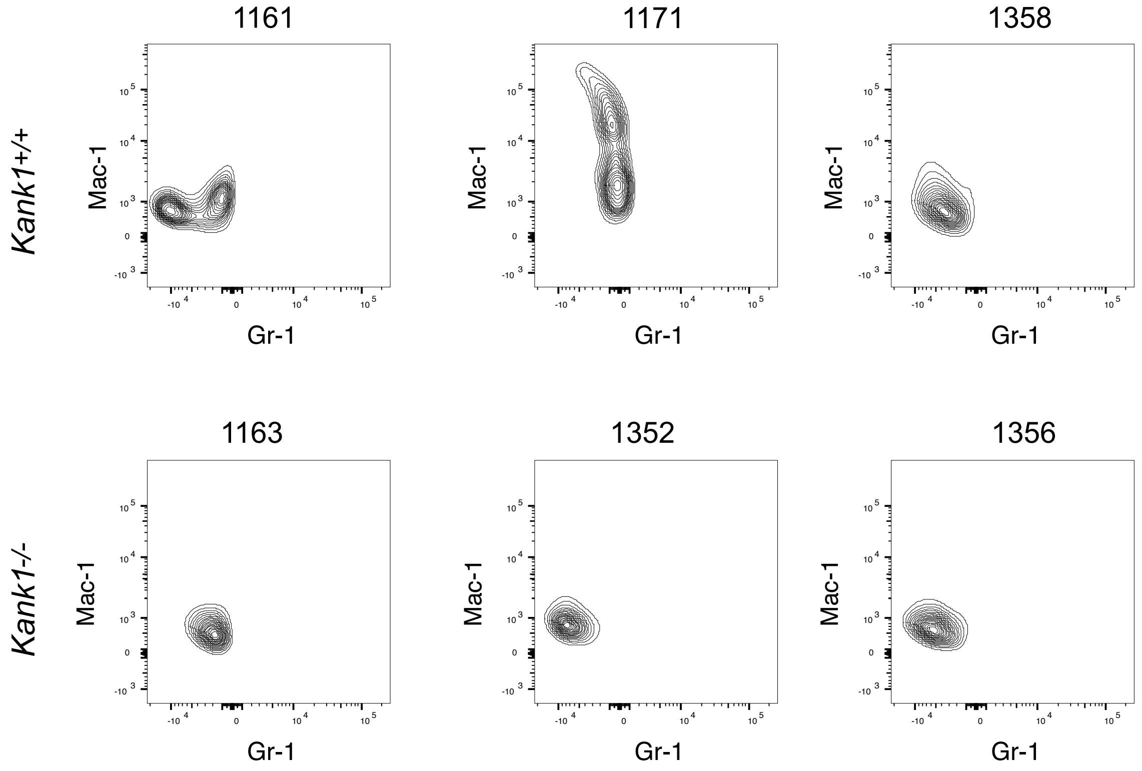

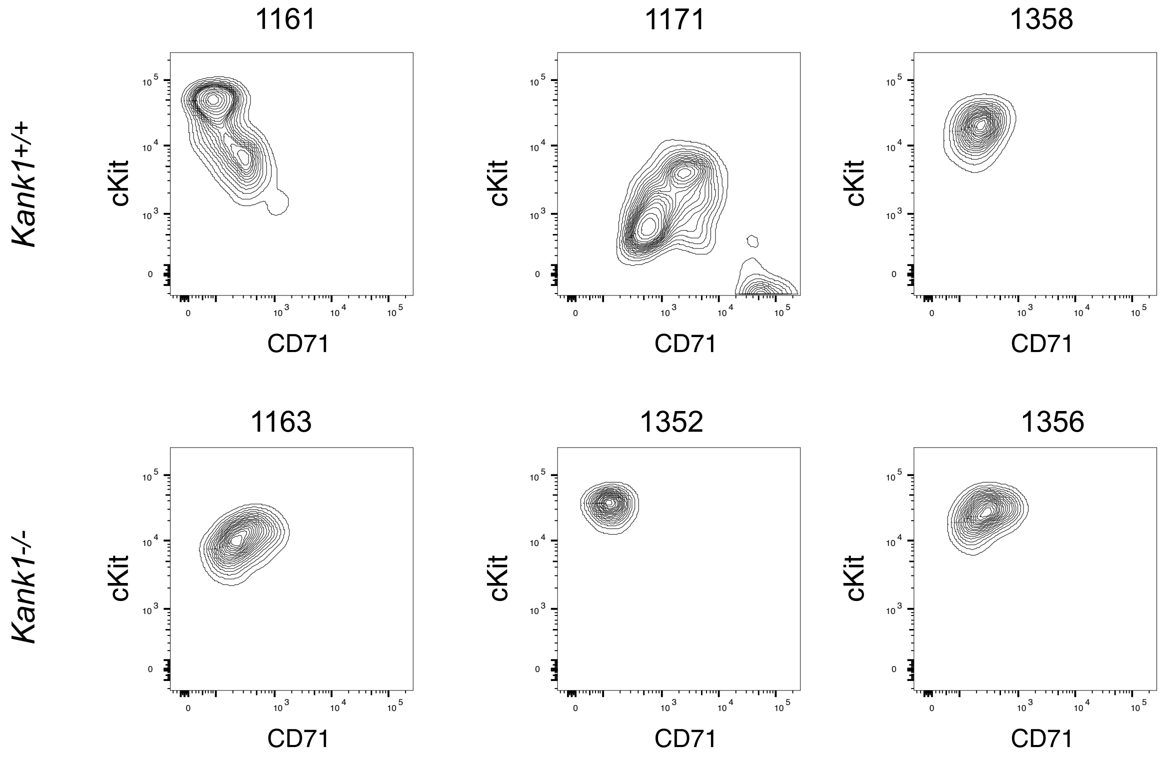

Supp. Figure S2

a

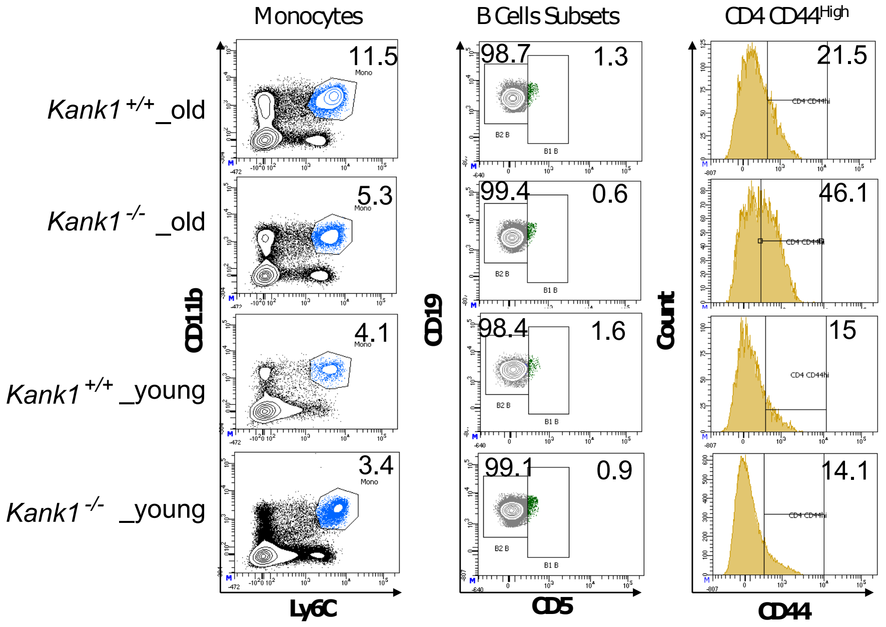

b

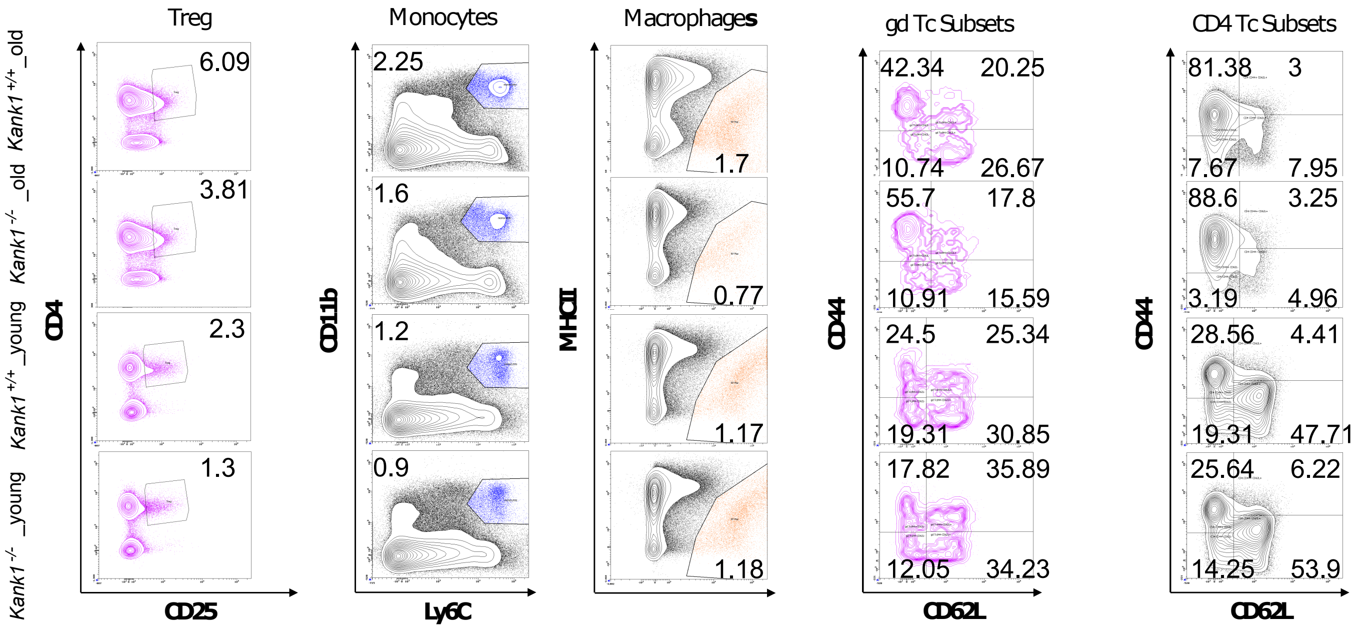

Supp. Figure S2

c

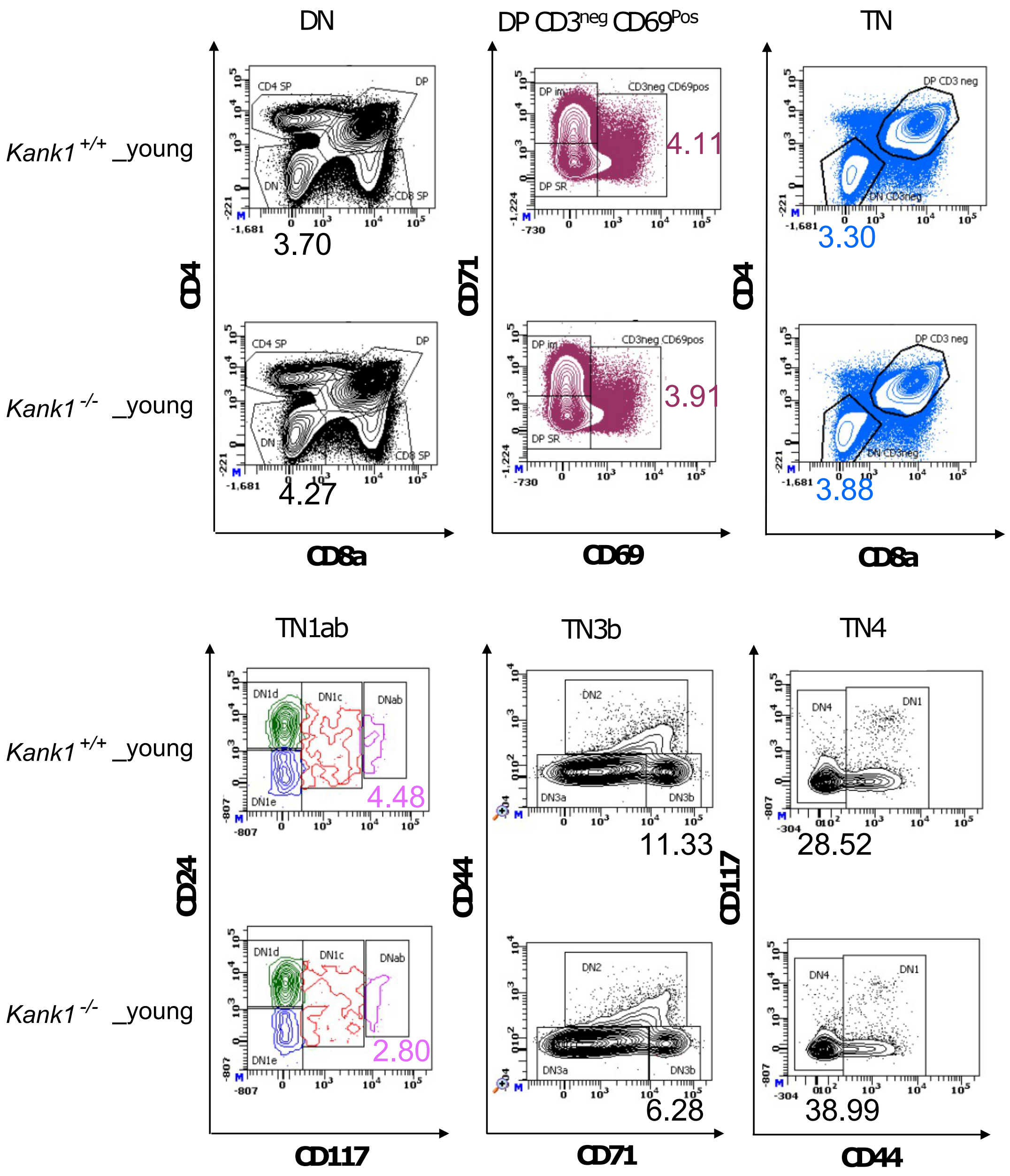

Supplement: Supplementary file 1 [file genes-14-01947-s001.zip › Supp.Materials_/Kank1-Supp-Fig-merged.pdf]
