# Supplementary material for: Loss of the KN Motif and AnKyrin Repeat Domain 1 (KANK1) Leads to Lymphoid Compartment Dysregulation in Murine Model
Source: Genes (Basel). 2023 Oct 16;14(10):1947. doi: 10.3390/genes14101947 (PMC10605996; doi:10.3390/genes14101947)
Supplement: Supplementary file 1 [file genes-14-01947-s001.zip › Supp.Materials_/Supp.Fig.legends.pdf]

Supplementary Figure S1: Characterization of *Kank1*$^{-/-}$ mouse model

a) Cytoscan HD SNP array analysis of blood from (**i**) patient, (**ii**) father, and (**iii**) mother showing a large loss-of-heterozygosity deletion of around 1.9Mbp at ch9p24.3: 535.417-725.581 (shaded in pink) which encompasses *KANK1* gene.
b) Agarose gel image of genotyping PCR products of *Kank1* locus using primers spanning the 22bp deleted sequence in exon5.
c) A schematic illustration (top panel) and relative expression (RE) q-PCR plots (lower panel) of *Kank1* mRNA expression in *Kank1*$^{+/+}$, *Kank1* $^{+/-}$ and *Kank1*$^{-/-}$ in liver cells. (**i**) Bar plot of relative expression (1/dCt) of *Kank1* mRNA using q-PCR primers overlapping the deleted gDNA sequence in exon 5 in *Kank1*$^{-/-}$ mice compared to *Kank1*$^{+/+}$ *and Kank1* $^{+/-}$. (**ii**) Bar plot of relative expression (1/dCt) of *Kank1* mRNA using q-PCR primers spanning exon 11-12 (n= 3 per genotype). Values are shown as relative expression normalized to *Gapdh*.
d) Bar plot of targeted mass spectrometry analysis of KANK1 protein expression levels in bone marrow (BM) cells from *Kank1*$^{+/+}$and *Kank1*$^{-/-}$ mice (n=3 per genotype).
e) Bar plots of various peripheral blood parameters including white blood cells (**WBC**), red blood cells (**RBC**), Hemoglobin (**HGB**) and platelets (**PLT**) of *Kank1*$^{+/+}$, *Kank1* $^{+/-}$ and *Kank1*$^{-/-}$ mice (n= 12 for *Kank1*$^{+/+}$ and *Kank1*$^{-/-}$ and n= 5 for *Kank1* $^{+/-}$).
f) Bar plots showing the quantification (%) of stem and progenitor cells population in the BM of adult (19 weeks) *Kank1*$^{+/+}$ and *Kank1*$^{-/-}$ mice (n=5 per genotype, n=4 females & n=1 male).
g) Bar plots showing the quantification (%) of stem and progenitor cells population in the BM of old (53-56 weeks) *Kank1*$^{+/+}$ and *Kank1*$^{-/-}$ mice (n=3 per genotype, n=2 females & n=1 male).
h) Flow cytometry plots showing the gating strategy for determining stem and progenitor cell populations.
i) Bar plots showing the quantification (%) of lineage cells population in the BM of adult (19 weeks) *Kank1*$^{+/+}$ and *Kank1*$^{-/-}$ mice (n=5 per genotype, n=4 females & n=1 male).
j) Bar plots showing the quantification (%) of lineage cells population in the BM of old (53-56 weeks) *Kank1*$^{+/+}$ and *Kank1*$^{-/-}$ mice (n=3 per genotype, n=2 females & n=1 male).
k) Flow cytometry plots showing the gating strategy for determining lineage cell populations.
l) Bar plot showing the quantification (%) of stem/erythroid markers, ckit and CD71 and myeloid markers Mac1 and Gr1 obtained from 40,000 total BM cells from *Kank1*$^{+/+}$ and *Kank1*$^{-/-}$ mice after 7 days in M3434 methylcellulose (n= 3 per genotype).

m) Flow cytometry plots showing the gating strategy and expression (%) of myeloid makers Mac1 & Gr1 (top panels) and stem/erythoid markers ckit and CD71 (lower panels) from first plating of 40,000 total BM cells from *Kank1*$^{+/+}$ and *Kank1*$^{-/-}$ mice (n= 3 per genotype).
n) Flow cytometry plots showing the expression (%) of myeloid makers Mac1 & Gr1 (top panels) and stem/erythoid markers ckit and CD71 (lower panels) from second plating of 40,000 total BM cells from *Kank1*$^{+/+}$ and *Kank1*$^{-/-}$ mice (n= 3 per genotype).
o) Flow cytometry plots showing the expression (%) of myeloid makers Mac1 & Gr1 (top panels) and stem/erythoid markers ckit and CD71 (lower panels) from third plating of 40,000 total BM cells from *Kank1*$^{+/+}$ and *Kank1*$^{-/-}$ mice (n= 3 per genotype).

Values are presented as individual points, bar graphs represent the mean value of biological replicates, error bars as standard error of the mean. Unpaired two-tailed t-test was used to calculate statistical significances.

Supplementary Figure S2: Comprehensive analysis of markers expression in *Kank1*$^{-/-}$ mice

a) Flow cytometry plots showing the gating strategy for peripheral blood cell markers analysis of old and young mice.
b) Flow cytometry plots showing the gating strategy for spleen cell markers analysis of old and young mice.
c) Flow cytometry plots showing the gating strategy for thymus cell markers analysis of young mice.

Values are presented as individual points, bar graphs represent the mean value of biological replicates, error bars as standard error of the mean. Statistical significances were tested with unpaired two-tailed t-test.

Supplementary Table S1: Antibody panels used for peripheral blood, spleen, and thymus comprehensive flow cytometry of old and young mice.
